# Beneficial Effect of Successful Simultaneous Pancreas-Kidney Transplantation on Plasma Profile of Metalloproteinases in Type 1 Diabetes Mellitus Patients

**DOI:** 10.3390/jcm10173800

**Published:** 2021-08-25

**Authors:** Jerzy Chudek, Aureliusz Kolonko, Jacek Ziaja, Tomasz Francuz, Dorota Kamińska, Aleksander J. Owczarek, Piotr Kuczera, Agata Kujawa-Szewieczek, Mariusz Kusztal, Adrian P. Kowalik, Dominika Bożek-Pająk, Joanna Kluz, Piotr Choręza, Robert Król, Magdalena Krajewska, Lech Cierpka, Andrzej Więcek

**Affiliations:** 1 Department of Internal Medicine and Oncological Chemotherapy, Medical University of Silesia, 40-027 Katowice, Poland; chj@poczta.fm; 2 Department of Nephrology, Transplantation and Internal Medicine, Medical University of Silesia, 40-027 Katowice, Poland; p.m.kuczera@gmail.com (P.K.); agata.szewieczek@gmail.com (A.K.-S.); awiecek@sum.edu.pl (A.W.); 3 Department of General, Vascular and Transplant Surgery, Medical University of Silesia, 40-027 Katowice, Poland; ziacek@wp.pl (J.Z.); adriankowalik@gmail.com (A.P.K.); dominikabp@mp.pl (D.B.-P.); rkrol@sum.edu.pl (R.K.); lcierpka@gan.pl (L.C.); 4 Department of Biochemistry, Medical University of Silesia, 40-752 Katowice, Poland; tfrancuz@sum.edu.pl; 5 Department of Nephrology and Transplantation Medicine, Wrocław Medical University, 50-556 Wrocław, Poland; dorotakaminska@interia.pl (D.K.); mariusz.kusztal@umed.wroc.pl (M.K.); magdalena.krajewska@umed.wroc.pl (M.K.); 6 Health Promotion and Obesity Management Unit, Department of Pathophysiology, Medical University of Silesia, 40-752 Katowice, Poland; aowczarek@sum.edu.pl (A.J.O.); pchoreza@sum.edu.pl (P.C.); 7 Department of Angiology, Hypertension and Diabetology, University Clinical Hospital, 50-556 Wrocław, Poland; joannakluz@wp.pl

**Keywords:** arterial stiffness, atherosclerosis, intima-media thickness, matrix metalloproteinases, simultaneous pancreas kidney transplantation, type 1 diabetes

## Abstract

It is not fully elucidated whether the restoring of normal glucose metabolism after successful simultaneous pancreas-kidney transplantation (SPK) improves vascular wall morphology and function in type 1 diabetic (T1D) patients. Therefore, we compared arterial stiffness, assessed by pulse wave velocity (PWV), carotid intima-media thickness (IMT), and biomarkers of arterial wall calcification in T1D patients after SPK or kidney transplantation alone (KTA). In 39 SPK and 39 KTA adult patients of similar age, PWV, IMT, circulating matrix metalloproteinases (MMPs) and calcification biomarkers were assessed at median 83 months post transplantation. Additionally, carotid plaques were visualized and semi-qualitatively classified. Although PWV and IMT values were similar, the occurrence of atherosclerotic plaques (51.3 vs. 70.3%, *p* < 0.01) and calcified lesions (35.9 vs. 64.9%, *p* < 0.05) was lower in SPK patients. There were significantly lower concentrations of MMP-1, MMP-2, MMP-3, and osteocalcin in SPK subjects. Among the analyzed biomarkers, only logMMP-1, logMMP-2, and logMMP-3 concentrations were associated with log HbA_1c_. Multivariate stepwise backward regression analysis revealed that MMP-1 and MMP-3 variability were explained only by log HbA_1c_. Normal glucose metabolism achieved by SPK is followed by the favorable profile of circulating matrix metalloproteinases, which may reflect the vasoprotective effect of pancreas transplantation.

## 1. Background

Simultaneous pancreas and kidney transplantation (SPK) is the treatment of choice in patients with type 1 diabetes (T1D) and end-stage renal disease. This procedure results in improved T1D patient survival as compared to kidney transplant alone (KTA) [1] even in case of those who received kidney graft from living donors [2,3]. Moreover, SPK patients have lower blood pressure, better left ventricular function, and better cardiovascular outcomes [4,5]. It is worth noting that both chronic kidney disease (CKD) and T1D are associated with increased arterial stiffness and progression of atherosclerosis in a time-dependent manner [6,7,8]. What appears important is that both large-vessel atherosclerosis and medial calcification are highly prevalent in this population and contribute to arterial wall dysfunction [6,7,9]. It was previously shown that microvascular damage, measured in the oral mucosa, is reversed early after successful SPK [10]. In our previous study, the endothelial function assessed by brachial flow-mediated dilation and plasma nitrites level was more improved in SPK recipients than in KTA patients [11]. However, both the arterial stiffness measured by pulse wave velocity (PWV) and the levels of serological markers of endothelial dysfunction were still elevated in a mid-term observation after successful SPK [12]. In observational studies, a slower progression of macrovascular disease was observed after SPK as compared with KTA, especially in the longer, 10-year follow-up period [13,14]. In line with that, the survival advantage attributed to the effect of restoring normoglycemia in T1D patients seems to be more likely demonstrated only in a sufficiently long follow-up period [15]. One could expect that the vasoprotective effect of the normoglycemia restoration should be detectable much earlier by analyzing markers of vascular remodeling in circulation. However, there are no data demonstrating whether long-term better glycemic control after successful SPK improves vascular wall morphology in T1D patients.

The matrix metalloproteinases (MMPs) family is a bundle of zinc-dependent proteases that are involved in the degradation and hydrolysis of extracellular matrix components [16]. Their activity contributes to all phases of vascular remodeling, especially in the diabetic and uremic milieu [16,17]. In T1D patients, some of MMPs were shown to be associated with arterial stiffness and pulse pressure [18] as well as cardiovascular events and all-cause mortality [19]. It has also been demonstrated that several MMPs may initiate vascular calcifications or accelerate this process [20,21].

The present study aimed to compare arterial stiffness, intima-media thickness, and several arterial wall calcification biomarkers as well as calcium-phosphate metabolism parameters in T1D patients long-term after SPK or KTA.

## 2. Methods

Here, we present the results of cross-sectional study in kidney transplant recipients, performed in two Polish transplant centers (in Katowice and Wrocław) between 2014 and 2018. The study protocol was approved by Bioethics Committee of the Medical University of Silesia in Katowice (KNW/0022/KB1/151/12), and each participant gave informed consent. No organs/tissues were procured from prisoners. All procedures were performed in accordance with the relevant guidelines and regulations. Clinical data were obtained from a database of transplant centers and from original medical records.

### 2.1. Study Group

Two groups of T1D patients after SPK (*n* = 39) or KTA (*n* = 39), with a minimum post-transplant follow-up period of 12 months, were enrolled into the study, as described previously [11]. In all patients, T1D requiring insulin therapy was diagnosed in childhood or adolescence, and CKD developed as a consequence of diabetic nephropathy. Both study groups did not differ in terms of recipient’s gender, BMI, duration of pre-transplant diabetes mellitus, and dialysis vintage, whereas the age difference was of borderline significance (*p* = 0.06).

The exclusion criteria [11] were as follows: a pre-emptive and living donor, second or third kidney transplantation, significantly impaired kidney graft function at the time of the study with estimated glomerular filtration rate (eGFR) ≤30 mL/min/1.73 m^2^ (calculated according to MDRD formula), bacterial infection within 1 month preceding the study, diagnosis of cancer or liver cirrhosis, apparent noncompliance, and additionally any type of antidiabetic medication currently used in SPK recipients.

In all patients, the kidney was transplanted on standard basis, and in SPK recipients, the pancreatic graft was placed intraperitoneally with systemic drainage of venous blood and enteric drainage of pancreatic juice.

### 2.2. Carotid Artery Intima-Media Thickness (IMT) and Carotid Plaques Assessment

Carotid ultrasound examination was performed using a Siemens machine (Sonoline Antares, Mountain View, CA, USA) equipped with a 4.0–9.0 MHz linear transducer. The evaluation comprised the common, internal, and external carotid arteries and the carotid bifurcation on each side. The common carotid artery IMT was measured proximal to the carotid bulb, omitting visible plaques. The results from three separate measurements on each side were then averaged. Additionally, at each examined location, the vessels were carefully evaluated concerning the presence of plaques, which were classified based on the following simplified scale: 0, no lesions; 1, non-calcified lesions; 2, at least one calcified lesion; 3, some calcified lesions; and 4, carotid bulb heavily covered with calcified lesions. The final plaque score was equal to the higher score obtained for both sides [22].

### 2.3. Carotid-Femoral Pulse Wave Velocity (PWV)

Arterial stiffness measurements were performed in the morning, after at least 15 min of rest in the supine position, using a non-invasive tonometer (Sphygmo-Cor 2000, AtCor Medical, Sydney, Australia) placed over the carotid and femoral arteries [22]. Pressure signals were calibrated using brachial blood pressure, and PWV was calculated as the time of pulse wave between the diagnosed points (distance (m)/time (s)).

### 2.4. Laboratory Measurements

The concentrations of blood-glycated hemoglobin (HbA_1c_), serum creatinine, lipids, calcium, and phosphate were routinely measured during standard visits in the out-patient clinic. Additionally, blood samples were collected from each patient in a closed system into tubes containing citrate and ethylenediaminetetraacetic acid (EDTA). The tubes were allowed to remain standing for 30 min at room temperature, then they were centrifuged (for 15 min., 3000 rpm) and finally preserved at −70 °C.

Enzyme-linked immunosorbent assays (ELISA) using commercially available kits were applied for: plasma concentration measurements of fetuin A (Biovendor, Modřice, Czech Republic; with limit of quantification (LoQ) of 0.1 ng/mL, intra-assay variation <3.6%, inter-assay variation <6.3%), high-sensitivity C-reactive protein—(CRP) (Immundiagnostic AG, Bensheim, Germany; with LoQ of 0.09 mg/L, intra-assay variation <6%, inter-assay variation <11.6%), osteoprotegerin (OPG) (Biovendor, Modřice, Czech Republic; with LoQ of 0.03 umol/L, intra-assay variation <4.9%, inter-assay variation <9.0%), matrix metalloproteinase 9 (MMP-9) (Biovendor, Modřice, Czech Republic; with LoQ of 0.05 ng/mL, intra-assay variation <7.3%, inter-assay variation <10.2%), fibroblast growth factor-23 (FGF23) (Immutopics, San Clemente, CA, USA, with intra-assay variation <4.4% and inter-assay variation <6.1%), and soluble α-Klotho (Immuno-Biological Laboratories Co. Ltd.; Fujioka-Shi, Gunma, Japan; with intra-assay variation <3.0% and inter-assay variation <6.5%). Plasma 25-hydroxylated vitamin D (25-OH-D) (LoQ 3 ng/mL) and intact parathyroid hormone (PTH) levels were assessed by electrochemiluminescence method (ECLIA) using commercially available kits in Cobas E411 analyzer (Roche Diagnostics GmbH, Mannheim, Germany) with inter-assay coefficients of variability of <7.8 and <6.5%, respectively.

Plasma levels of matrix metalloproteinases (MMP-1, MMP-2, and MMP-3), osteocalcin (OC), and osteopontin (OP) were measured using multiplex Bio-Plex kits (Bio-Rad, CA, USA), in accordance with the manufacturer’s instructions. Median bead fluorescence readings were taken using the Bio-Plex 200 System with high PMT (High RP1) setting and analyzed with Bio-Plex Manager version 6.1.0.727 (Bio-Rad Laboratories, Hercules, CA, USA).

### 2.5. Data Analysis

Blood pressure (BP) was measured at the beginning of study: three times in the sitting position at the arm without arterio-venous fistula. Patients with BP values above 140/90 mmHg or those who received antihypertensive medication were diagnosed as hypertensive.

Vitamin D status was categorized by commonly used cut-offs and definitions of serum 25-OH-D: the values below 10 ng/mL were categorized as severely deficient, between 10 and 19.9 ng/mL as deficient, between 20 and 29.9 ng/mL as insufficient, and those with values equal to or above 30 ng/mL as sufficient 25-OH-D concentrations [23].

The kidney graft function was measured using eGFR calculated according to MDRD formula.

### 2.6. Statistical Analysis

Statistical analysis was performed using Statistica 13.3 PL for Windows (Tibco Inc., Palo Alto, CA, USA), StataSE 12.0 (College Station, TX, USA), and R software (version 4-0-5, The R Project for Statistical Computing, https://www.r-project.org) (accessed on 3 August 2021). The level of statistical significance was set at *p* value below 0.05. All tests were two-tailed. Imputations were not performed for a few missing clinical data. Nominal and ordinal data were expressed as percentages, whilst interval data were expressed as mean value ± standard deviation (SD) in case of normal distribution or as median with lower and upper quartile (1-3Q) in case of data with skewed or non-normal distribution. The distribution of variables was evaluated by the Shapiro–Wilk test, and the homogeneity of variances was assessed by Levene test.

To compare data between analyzed groups, Student’s *t*-test was used. In case of skewed distribution, normalization with logarithmic function was performed. Categorical variables were compared using either χ^2^ tests or χ^2^ tests with Yates correction.

In order to assess the relationship between variables, linear regression was used. Multivariate models for the variability of MMP-1, MMP-2, MP-3, and OC included potential independent variables selected on the basis of univariable regression analyses, i.e., eGFR, log_10_(HbA_1c_), log_10_(CRP), HDL, and log_10_(TG). Cook–Weisberg test and Cameron and Trivedi’s decomposition test were used to test the residuals for heteroscedasticity as well as the violation of skewness and kurtosis assumptions in linear regression. The correlation between variables was assessed either with Pearson linear correlation coefficient or Spearman rank correlation coefficient, depending on data distribution.

## 3. Results

### 3.1. Study Groups Characteristics

The characteristics of SPK and KTA groups are given in Table 1. There was no significant difference in post-transplant follow-up period between these groups (Me: 80 (1-3Q: 67–110) vs. Me: 86 (1-3Q: 47–106) months, respectively; NS). As expected, SPK patients showed markedly lower HbA_1c_ levels. In addition, the proportion of patients with HbA_1c_ in the reference range for healthy individuals (below 5.5%) was significantly higher in SPK than in KTA group (61.5 vs. 0%, respectively; *p* < 0.001). Patients in SPK group had higher eGFR values. Of note, SPK patients were more frequently receiving tacrolimus-based, steroid-free regimens (Table 1) and received organs from significantly younger donors (24 (95%CI: 22–26) vs. 44 (40–49) years). In addition, this group presented a slightly but significantly higher high-density lipoprotein (HDL)-cholesterol level (not corresponding with the frequency of statin usage) and lower level of triglycerides (TG). The plasma levels of CRP were comparable in both study groups.

We found higher serum concentration of PTH and total serum calcium in patients in KTA group, whereas serum phosphorus level and plasma concentrations of 25-OH-D, α-Klotho, and FGF-23 were similar in both groups (Table 1). The observed differences cannot be explained by lower use of alphacalcidol in KTA patients.

### 3.2. Structural and Functional Measures of Vascular Injury

Ultrasound imaging of carotid arteries revealed a lower occurrence of atherosclerotic plaques (51.3 vs. 70.3%; *p* < 0.01), especially calcified lesions (35.9 vs. 64.9%; *p* < 0.05), in the SPK subjects. Nevertheless, IMT and PWV values were similar in both groups (Table 1).

The patients with calcified lesions (*N* = 48; 20 in SPK and 28 in KTA) were significantly older (50.9 ± 7.8 vs. 43.5 ± 7.3 years; *p* < 0.001) and were characterized by greater IMT (Me: 0.8 (1-3Q: 0.7–0.9) vs. Me: 0.7 (1-3Q: 0.6–0.7) mm; *p* < 0.001) than the subjects without calcified lesions. Additionally, they were more likely to use statins (42.1 vs. 18.4%; *p* < 0.05). Of note, the difference in median HbA_1c_ concentration (Me: 50 (1-3Q: 38–66) in SPK vs. Me: 42 (1-3Q: 33–60)% in KTA; *p* = 0.08) and eGFR (61.9 ± 26.1 in SPK vs. 52.5 ± 21.6 mL/min/1.73m^2^ in KTA; *p* = 0.09) between the study groups did not reach statistical significance. Similarly, there was no significant difference in OC levels between these groups (Me: 2268 (1-3Q: 1253–3525) vs. 1339 (1-3Q: 828–3034) pg/L, *p* = 0.31). There was a significant positive correlation between increasing plaque score and age (τ = 0.402; *p* < 0.001). The subjects with plaque score ≥ 3 were more likely to use statins (N: 12 (52.2%) vs. 11 (20.7%); *p* < 0.01). Nevertheless, there was no significant association between plaque score and eGFR and OC, metalloproteinases, or HbA_1c_ levels.

### 3.3. Biomarkers of Vascular Injury

Among the circulating markers of vascular wall remodeling, we found significantly lower values of MMP-1, MMP-2, MMP-3, and OC in SPK subjects. In contrast, there were no differences in MMP-9, fetuin-1, OPG, and OP levels (Table 1).

**Figure 1:**
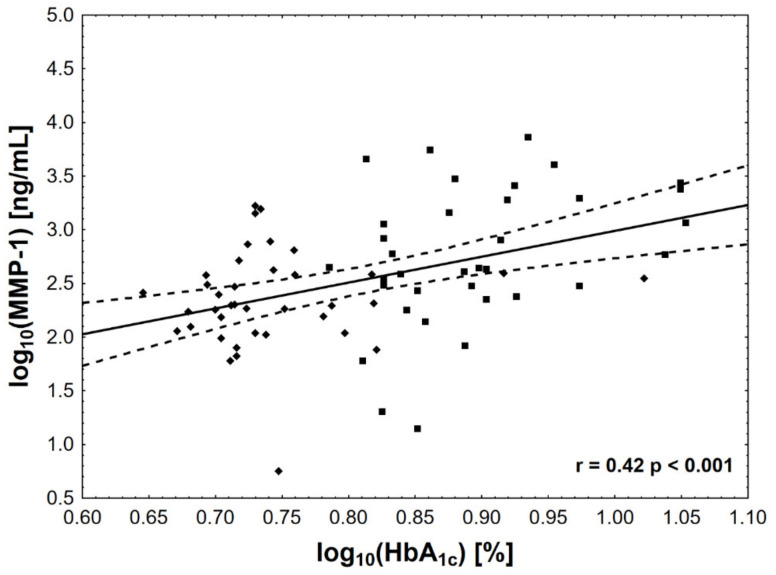
The associations between HbA_1c_ concentration and MMP-1 activity. The value of log HbA_1c_ of 0.78 corresponds to HbA_1c_ level = 42 mmol/mol (6.0%), log HbA_1c_ of 0.9—to HbA_1c_ level = 64 mmol/mol (8.0%), and log HbA_1c_ of 1.0—to HbA_1c_ level = 86 mmol/mol (10%).

**Figure 2:**
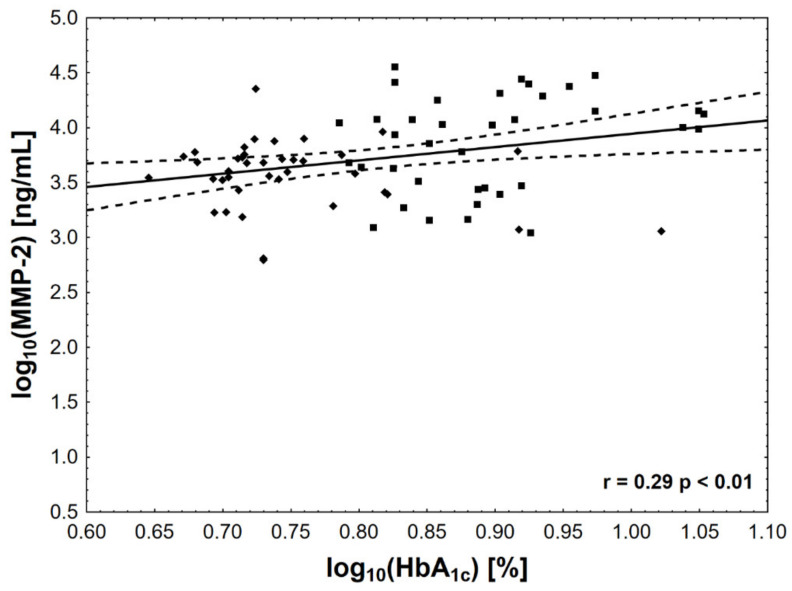
The associations between HbA_1c_ concentration and MMP-2 activity. The value of log HbA_1c_ of 0.78 corresponds to HbA_1c_ level = 42 mmol/mol (6.0%), log HbA_1c_ of 0.9—to HbA_1c_ level = 64 mmol/mol (8.0%), and log HbA_1c_ of 1.0—to HbA_1c_ level = 86 mmol/mol (10%).

**Figure 3:**
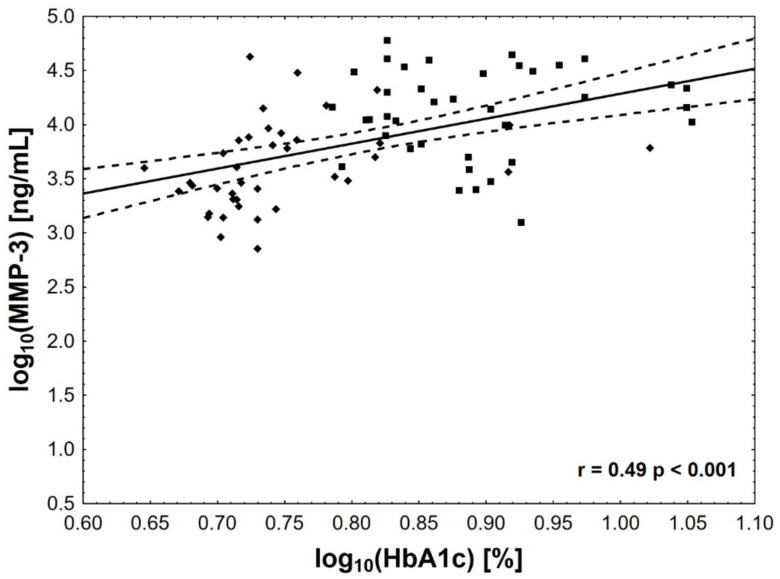
The associations between HbA_1c_ concentration and MMP-3 activity. The value of log HbA_1c_ of 0.78 corresponds to HbA_1c_ level = 42 mmol/mol (6.0%), log HbA_1c_ of 0.9—to HbA_1c_ level = 64 mmol/mol (8.0%), and log HbA_1c_ of 1.0—to HbA_1c_ level = 86 mmol/mol (10%).

Of all the analyzed biomarkers, only log MMP-1 (Figure 1), log MMP-2 (Figure 2), and log MMP-3 (Figure 3) concentrations were associated with log HbA_1c_ (Table 2). In addition, both log MMP-1 and log MMP-3 were inversely related to eGFR. Log MMP-1 also correlated with log CRP, log TG, and statin-adjusted HDL levels, whereas log MMP-3 correlated with log CRP and log TG (Table 2). Of note, log OPG and log OC were inversely associated only with eGFR. We did not find significant correlations for OP. Notably, there was also a correlation between plasma osteocalcin and PTH concentrations (ρ = 0.33, *p* < 0.01).

The multivariate stepwise backward regression analysis revealed that variabilities of MMP-1 and MMP-3 values were explained by log HbA_1c_, whereas OC variability only by eGFR (Table 3).

## 4. Discussion

The main finding of our present study is the demonstration of significantly lower concentration of circulating metalloproteinases (MMP-1, MMP-2, and MMP-3) in SPK recipients as compared with KTA patients, which potentially may reflect the beneficial changes in the vascular wall related to the normalization of glucose metabolism. Importantly, multivariate analysis confirmed the independent association of HbA_1c_ levels with MMP-1 and MMP-3 concentrations. Additionally, SPK subjects also presented with lower plasma osteocalcin level, but it was related merely to better kidney graft function.

It has been postulated that restoring of normoglycemia after successful SPK transplantation should be followed by favorable effect on the vascular system, which will compensate the increased risk of surgical and infectious complications, including death, related to combined organ transplantation. However, taking into account the advanced vascular wall damage already present at the time of transplantation as a consequence of several decades of diabetes and subsequent uremia, the expected vascular improvement after SPK is difficult to demonstrate in cohort studies. Additionally, the lack of such evidence can also be explained by non-optimal metabolic efficiency in a significant proportion of transplanted pancreas, which may result from their fragmentary thrombosis and even more often from weight gain and increasing insulin resistance after transplantation [24,25]. In line with the above, in the present study, the surrogate of arterial stiffness (PWV) and carotid atherosclerosis were shown to be similar in SPK and KTA patients. Of note, Larsen et al. previously showed that two years after successful pancreas transplantation in T1D patients, IMT values decreased significantly along with optimal glucose metabolism control [26]. Nonetheless, the analysis of vascular wall remodeling biomarkers showed significant associations between HbA_1c_ and MMP-1, MMP-2, and MMP-3 plasma levels, confirmed by multivariate analysis for MMP-1 and MMP-3. This indirect evidence supports the expected improvement in vascular wall metabolism related to normal glucose metabolism control in SPK patients.

MMP-2 and MMP-9 are considered to be main enzymes that degrade collagen type IV, the key component of extracellular matrix that determines the structure of vessels and glomerular base membranes [27]. Previously, the upregulation of MMP-2 and MMP-9 was shown in arterial vasculature of diabetic CKD patients and turned out to be correlated with arterial stiffness [28], whereas a MMP-3 gene polymorphism (rs3025058) was associated with subclinical markers of coronary atherosclerosis and IMT progression in type 2 diabetes [29]. Moreover, several advanced glycation end-products were associated with plasma levels of MMP-2 and MMP-3 in T1D individuals [30]. Notably, in line with our present findings, Reine et al. described beneficial effects of successful SPK as compared to KTA on the kidney graft extracellular matrix structure in approximately 10-year observation in T1D patients [31]. We may hypothesize that the long-term restoration of normoglycemia results in favorable MMPs profile, which probably reflects the slowing of vascular system damage (diabetic vasculopathy/remodeling). Our finding could be supported by long-term observational studies analyzing the evolution of vascular function parameters during more than 10-year follow-up period after SPK.

When considering the potential association between the metabolism of glucose and established biochemical markers of vascular calcification, none of them turned out to be associated with HbA_1c_ level. Although plasma osteocalcin levels were shown to be significantly lower in SPK patients, it was attributed to better kidney graft function and lower serum PTH concentration but not to HbA_1c_ blood level in this group. To date, the only study in small insulin-independent SPK cohort reported mildly elevated osteocalcin levels in 45% of the patients, with similar association with PTH [32]. Previously, osteocalcin has been found in calcified atherosclerotic plaques [33]. On the other hand, OC has been shown to stimulate insulin secretion by pancreas and glucose uptake in the adipose tissue [34]. Nonetheless, the utility of OC as a biomarker is still discussed in conflicting studies [34].

The main limitation of this study is its low number of participants. However, we included all eligible SPK patients attending our out-patient clinic who gave their consent to participate in the research. Additionally, we recruited the KTA group based on two transplant centers due to the low number of T1D patients in whom KTA was performed. Moreover, in the effort to improve the study groups match in comparison to our original analysis [11], especially with regard to age and the post-transplant follow-up period, we reassessed the eligible SPK subjects again and repeated all biochemical parameters using the same methodology. It should be stressed that the enrollment of T1D patients after KTA as a control group eliminates the bias of potential metabolic memory related to uremic milieu.

The list of study limitations is longer. At the time of the study, we have noted significant differences in immunosuppression regimens and lipid levels between the examined groups that may interfere with the study results. In fact, patients in SPK group were exclusively treated with tacrolimus-based, predominantly steroid-free regimens, whereas a relevant part of KTA patients were treated with cyclosporine A and prednisone. As both tacrolimus and steroids affects glucose metabolism, the net effect of immunosuppressive regimen on vascular wall damage is at least partially balanced. Furthermore, the independent effect of lipid levels on circulating MMPs was not supported in multivariable analyses, which suggests that their influence is smaller than that of the normal glucose metabolism restored with SPK.

In conclusion, in our study we demonstrated that SPK recipients are characterized by a favorable profile of circulating matrix metalloproteinases in comparison to KTA subjects with T1D. This potential vasoprotective effect of SPK transplantation seems to be mediated mainly by the normalization of glucose metabolism.

## Figures and Tables

**Table 1:** Basic characteristics of both study groups (SPK and KTA).

	SPK	KTA	*p*
	*n* = 39	*n* = 39	
Sex (male) (*n* (%))	17 (43.6)	17 (43.6)	1.00
Age at the time of study (years)	44 (40–51)	49 (42–56)	0.06
Total duration of diabetes (years)	26 ± 6	29 ± 7	0.10
Duration of dialysis therapy prior transplant (months)	17.0 (11.0–27.0)	23.5 (14.0–35.0)	0.13
Follow-up after transplant (months)	87.9 ± 36.1	84.5 ± 41.0	0.70
Current immunosuppression:			
Tacrolimus (*n* (%))	39 (100)	20 (51.3)	<0.001
Tacrolimus level (*n*g/nL)	5.5 ± 1.3	5.6 ± 1.4	0.71
Cyclosporin A (*n* (%))	0	19 (48.7)	<0.001
Cyclosporine A (ng/mL)	-	92.5 ± 19.9	-
Antimetabolite drugs (*n* (%))	33 (84.6)	37 (94.9)	0.14
Everolimus (*n* (%))	6 (15.4)	1 (2.6)	<0.05
Prednisolone (*n* (%))	4 (10.3)	25 (64.1)	<0.001
Prednisolone daily dose (mg/day)	5.0 (5.0–5.0)	5.0 (5.0–5.0)	0.36
BMI (kg/m^2^)	22.6 ± 3.0	23.8 ± 3.6	0.11
Active smokers (*n* (%))	6 (15.4)	0	<0.05
HbA_1c_ (%)	5.37 (5.06–5.75)	7.70 (6.70–8.43)	<0.001
HbA_1c_ (mmol/mol)	35 (32–39)	61 (50–69)	<0.001
eGFR (mL/min/1.73 m^2^)	62.9 ± 24.1	48.7 ± 16.4	<0.01
Total cholesterol (mmol/L)	4.96 ± 1.06	5.21 ± 1.01	0.30
LDL-cholesterol (mmol/L)	3.18 ± 0.80	3.10 ± 1.04	0.72
HDL-cholesterol (mmol/L)	1.69 ± 0.39	1.50 ± 0.42	<0.05
Triglycerides (mmol/L)	0.91 (0.50–1.25)	1.33 (1.07–1.70)	<0.05
Statins (*n* (%))	8 (20.5)	15 (38.5)	0.08
Total calcium (mmol/L)	2.35 ± 0.13	2.41 ± 0.14	<0.05
Phosphorus (mmol/L)	1.11 ± 0.26	1.08 ± 0.18	0.38
FGF23 (pg/mL)	39.43 (18.34–70.19)	28.84 (16.26–57.56)	0.15
α-Klotho (pg/mL)	728 (507–905)	864 (578–1080)	0.51
PTH (pg/mL)	47.7 (30.1–85.1)	67.8 (51.9–96.3)	<0.05
25-OH-D (ng/mL)	6.68 (4.36–11.96)	9.18 (4.65–13.41)	0.48
25-OH-D < 10 (*n* (%))	26 (66.67)	23 (60.53)	0.58
Alphacalcidol use (*n* (%))	18 (48.7)	2 (5.1)	<0.001
CRP (mg/L)	1.2 (0.3–3.0)	1.4 (0.5–5.6)	0.26
Fetuin-1 (ng/mL)	22.8 (19.7–26.3)	20.8 (16.4–25.1)	0.10
Osteoprotegerin (pmol/L)	2.35 (0.93–3.59)	1.90 (1.34–3.05)	0.96
Osteocalcin (ng/mL)	1.31 (0.8–2.04)	2.58 (1.34–3.74)	<0.01
Osteopontin (ng/mL)	5.8 (4.24–7.73)	5.84 (4.18–7.89)	0.88
MMP-1 (ng/mL)	0.20 (0.11–0.38)	0.45 (0.27–1.97)	<0.01
MMP-2 (ng/mL)	4.04 (2.59–5.66)	10.44 (2.99–17.88)	<0.01
MMP-3 (ng/mL)	3.67 (2.05–7.24)	14.52 (6.72–30.95)	<0.001
MMP-9 (ng/mL)	88.4 (64.5–183.4)	121.5 (96.6–160.6)	0.46
Carotid plaques (*n* (%))			0.01
Absent	19 (48.7)	11 (29.7)	
Non-calcified	6 (15.4)	2 (5.4)	
Sole calcified lesion	7 (18.0)	8 (21.6)	
Few calcified lesions	2 (5.1)	5 (13.5)	
Massive calcified lesions	5 (12.8)	11 (29.7)	
IMT (mm)	0.73 ± 0.15	0.77 ± 0.13	0.30
PWV (m/s)	12.1 ± 4.1	11.1 ± 3.9	0.30

Data presented as numbers and frequencies or mean ± standard deviation or median (lower quartile—upper quartile) if appropriate. SPK, simultaneous pancreas-kidney transplantation; KTA, kidney transplantation alone; BMI, body mass index; HbA1c, glycated hemoglobin; eGFR, estimated glomerular filtration rate; LDL, low density lipoprotein; HDL, high-density lipoprotein; FGF−23, intact fibroblast growth factor 23; PTH, intact parathormon; 25-OH-D, 25-hydroxylated vitamin D; CRP, high sensitive C-reactive protein; MMP, metalloproteinase; IMT, intima-media thickness; PWV, pulse wave velocity.

**Table 2:** Factors influencing concentrations of circulating markers of vascular remodeling—univariate linear regression analyses.

Parameter	log_10_(HbA_1c_) (%) r	Cholesterol (mmol/L) r	LDL (mmol/L) r	HDL * (mmol/L) r	log_10_(TG) * (mmol/L) r	CRP (mg/L) r	eGFR (mL/min/1.73 m^2^) r
log_10_(Osteoprotegerin (pmol/L))	-	-	-	-	-	-	−0.30 *p* < 0.01
log_10_(Osteocalcin) (ng/mL)	-	-	-	-	-	-	−0.37 *p* < 0.01
Osteopontin (ng/mL)	-	-	-	-	-	-	-
log_10_(MMP-1) (ng/mL)	0.42 *p* < 0.001	-	-	0.33 *p* < 0.05	0.36 *p* < 0.01	0.23 *p* = 0.05	−0.36 *p* < 0.01
log_10_(MMP-2) (ng/mL)	0.30 *p* < 0.01	-	-	-	-	-	-
log_10_(MMP-3) (ng/mL)	0.49 *p* < 0.001	-	-	-	0.38 *p* < 0.01	0.26 *p* < 0.05	−0.38 *p* < 0.001
log_10_(MMP-9) (ng/mL))		-	-	-	-	-	-

* Statin-adjusted. HbA1c, glycated hemoglobin; LDL, low density lipoprotein cholesterol; HDL, high-density lipoprotein cholesterol; TG, triglycerides; CRP, high sensitive C-reactive protein; eGFR, estimated glomerular filtration rate; r, correlation coefficient; MMP, metalloproteinase.

**Table 3:** Factors influencing concentrations of circulating markers of vascular remodeling- stepwise backward regression analysis.

	β SE(β)				
Parameter	log_10_(MMP-1) (ng/mL)	log_10_(MMP-2) (ng/mL)	log_10_(MMP-3) (ng/mL)	log_10_(Osteocalcin) (ng/mL)	log_10_(Osteoprotegerin) (pmol/L)
eGFR (10 mL/min/1.73 m^2^)	-	-	-	−0.060 (0.017) *p* < 0.001	-
log_10_(HbA_1c_) (%)	2.415 (0.614) *p* < 0.001	-	2.304 (0.473) *p* < 0.001	-	-
log_10_(CRP) (mg/L)	-	-	-	-	-
HDL * (mmol/)	-	-	-	-	-
log_10_(TG) * (mmol/L)	-	-	-	-	-

* Statin-adjusted. MMP, metalloproteinase; eGFR, estimated glomerular filtration rate; HbA1c, glycated hemoglobin; CRP, high sensitive C-reactive protein; TG, triglycerides.

## Data Availability

The data presented in this study are available on request from the corresponding author. The data are not publicly available due to privacy.
